# High-Throughput Strategy for Profiling Sequential Section With Multiplex Staining of Mouse Brain

**DOI:** 10.3389/fnana.2021.771229

**Published:** 2021-12-23

**Authors:** Siqi Chen, Zhixiang Liu, Anan Li, Hui Gong, Ben Long, Xiangning Li

**Affiliations:** ^1^Britton Chance Center for Biomedical Photonics, Wuhan National Laboratory for Optoelectronics, Huazhong University of Science and Technology, Wuhan, China; ^2^HUST-Suzhou Institute for Brainsmatics, JITRI, Suzhou, China; ^3^Key Laboratory of Biomedical Engineering of Hainan Province, School of Biomedical Engineering, Hainan University, Haikou, China

**Keywords:** serial sections, registration, multiplex staining, high-throughput, mouse brain

## Abstract

The brain modulates specific functions in its various regions. Understanding the organization of different cells in the whole brain is crucial for investigating brain functions. Previous studies have focused on several regions and have had difficulty analyzing serial tissue samples. In this study, we introduced a pipeline to acquire anatomical and histological information quickly and efficiently from serial sections. First, we developed a serial brain-slice-staining method to stain serial sections and obtained more than 98.5% of slices with high integrity. Subsequently, using the self-developed analysis software, we registered and quantified the signals of imaged sections to the Allen Mouse Brain Common Coordinate Framework, which is compatible with multimodal images and slant section planes. Finally, we validated the pipeline with immunostaining by analyzing the activity variance in the whole brain during acute stress in aging and young mice. By removing the problems resulting from repeated manual operations, this pipeline is widely applicable to serial brain slices from multiple samples in a rapid and convenient manner, which benefits to facilitate research in life sciences.

## Introduction

As the most complex organ in mammals, the brain is comprised of billions of cells (Zhao et al., 2020). It is these cells with thousands of distinct types that interact with each other and forms a highly interconnected network, which provides consciousness and behavior (Craddock et al., 2013; Helmstaedter, 2013; Oh et al., 2014). To explore how these cells are organized together, researchers need to investigate the genotyping, distribution, and connections of different types of cells in each brain region. As even a single behavior requires the cooperation of many brain regions, including the whole brain, while the anatomical functional units are different in these regions, it is essential to obtain the structural information at the whole tissue scale rather than relying on a given area or adjacent region.

Significant efforts have been made in the past decade to map large numbers of neurons to better understand the network in the whole brain (Economo et al., 2016; Ni et al., 2021; Zhong et al., 2021). Recently, there has been an increasing interest in optimizing methods for volume imaging of molecularly labeled structures in large, cleared tissue samples (Pan et al., 2016; Park et al., 2018; Cai et al., 2019; Qi et al., 2019). However, the complex sample preparation, low efficiency, and high cost may remain a problem for researchers. Meanwhile, large-scale 3D imaging methods, such as optimized serial two-photon tomography (Ragan et al., 2012; Renier et al., 2016), also provide a good idea to overcome the whole-brain mapping of many animals. However, it requires significant resources, resulting in difficulties for laboratory use. Traditionally, 3D information of fixed brains and other biological samples was obtained by reconstructing a series of 2D images of mechanically cut thin sections (Gleave et al., 2013; Yan et al., 2018). To date, histological sectioning and staining remain the most popular methods for evaluating and identifying the location of specific proteins for both research and diagnostic purposes (van der Loos, 2007; Prevedel et al., 2014; Zhanmu et al., 2019).

In this study, we established a pipeline for processing serial slices of a complete mouse brain, including the physical experimental equipment and image processing software. To stain serial slices, we employed a conventional 24-well plate combined with a homemade section staining box, improving the slice integrity rate to 98.5%. For cell identification and registration, we utilized a custom program to realize the semiautomatic slice registration and automatic cell recognition and mapping. To demonstrate the application of the computational pipeline to the high-throughput brain-slice processing, we investigated the immediate early gene responses in normal and aging mice under stress conditions and indicated the potential weak-responding brain areas in aging mice.

## Materials and Methods

### Forced Swimming Test

Each mouse was placed in a 5 L glass beaker containing 3 L warm water at 25°C for 5 min, and a video camera was used to record the behavior of the mouse in real time (Castagné et al., 2011; Fitzgerald et al., 2020; Hu et al., 2020). After swimming, the mouse was removed from the glass beaker, dried in a heater, and placed in a cage to rest. After 90 min of the forced swimming test (FST), the mice were anesthetized and perfused. Subsequently, batch slice acquisition and immunohistochemistry were performed as described below, and whole-brain serial slices were stained with antibodies.

### Tissue Preparation

Adult male C57BL/6J mice were used in this study and were housed in normal cages in an environment with a 12 h light/dark cycle with food and water *ad libitum*. All animal experiments were approved by the Animal Ethics Committee of the Huazhong University of Science and Technology. Eighteen C57BL/6J mice were tested using the FST. Nine mice at 2–3 months were divided into a normal group and nine mice at 20 months belonging to the aging group. In addition, another two adult mice were selected as the control group without FST. Then, we randomly chose four mice in each normal group and aging group for immunostaining experiments to evaluate the response brain area and cell type in the FST experiment.

All histological procedures were conducted as follows: the mice were anesthetized by intraperitoneal injection with 1% sodium pentobarbital solution and, subsequently, intracardially perfused with 0.01 M phosphate-buffered saline (PBS, Sigma-Aldrich, St. Louis, MO, United States, P3813), and then followed by 4% paraformaldehyde (PFA, Sigma-Aldrich, St. Louis, MO, United States, 158127) in 0.01 M PBS. The brains were then excised and postfixed in 4% PFA at 4°C for 24 h. After fixation, each intact brain was rinsed overnight at 4°C in 0.01 M PBS.

### Acquisition of Batch Serial Tissue Section

**FIGURE 1:**
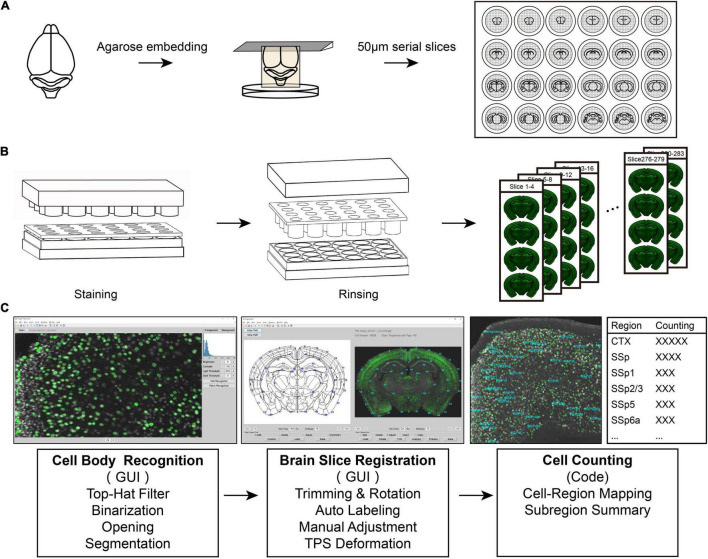
An integrated pipeline for processing batch tissue sections. **(A)** The procedure of acquiring continuous batch slices. The samples are embedded and sectioned in agarose. **(B)** The staining process for batch sections. Using the fitting and separation of the 24-well plate, the staining plate may efficiently realize the rapid processing of batch slices. **(C)** Diagram of registration and signal recognition.

The prepared mouse brain was embedded in agarose, as previously described (Jiang et al., 2017). The brains were dried and embedded in melted oxidized agarose using a silicone mold. There were several cuboid-shaped grooves in the mold for brain embedding and gridlines for correcting the brain orientation. The mold and brains were placed in a 55°C water bath for 0.5 h until the surfaces of the brains were fully coated with agarose. During the water bath, the orientation of the brain could be easily adjusted to ensure the correct sectioning angle of the whole brain. Then, the mold and brains were left at room temperature for 0.5 h to allow the agarose to solidify. The embedded mouse brain samples were glued to the sample base of a vibratome (VT 1200S; Leica, Wetzlar, Germany). By adjusting the amplitude to 1.0 mm and the slicing speed to 1.5 mm/s, the slices were cut to a thickness of 50 μm (Figure 1A).

### Batch Immunostaining

The collected serial sections of the whole brain were used for subsequent staining. A stained plate containing serial brain slices was placed in a 24-well plate. The homemade 24-well plate system contained a 24-well plate for storing the dyeing solution with a staining plate to place brain slices. This configuration guaranteed that the upper and lower surfaces of brain slices could be immersed into the dyeing solution (Supplementary Figure 1). We lifted the staining plate with the strainer and replaced the solution in a 24-well plate with other solutions. Using this methodology, we replenished all the rinsing and staining solutions for the batch tissue sections. The prepared slices were washed using PBS for 3 × 10 min, permeabilized using PBST (0.3% Triton-X-100 in PBS) for 1 h, and then incubated with blocking solution [5% bovine serum albumin (BSA) in PBST] for 1 h followed by incubation with primary antibodies overnight at 4°C. After washing the slices for 3 × 10 min in PBS, the appropriate secondary antibodies were applied for 2 h at room temperature. Finally, we used antifluorescence attenuation mounting tablets to mount the slides and inverted the confocal microscope (Zeiss LSM710, Oberkochen, Germany) and multichannel fluorescence slide microscope (Olympus VS120, Tokyo, Japan) for imaging.

To achieve a low damage rate and uniform staining effect of the batch tissue slices, we utilized one adult mouse brain to acquire 50 μm batch slices and performed immunostaining with NeuN antibody (Weyer and Schilling, 2003). The following antibodies were used: mouse anti-NeuN (BioLegend, San Diego, CA, United States, SIG-39860, 1:800 dilution) and Alexa Fluor 488 goat antimouse immunoglobulin G (IgG) antibody (Invitrogen, Carlsbad, CA, United States, A11029, 1:1,000 dilution). To verify the integrity of the brain slices after batch staining, the 158th brain slice was selected for counterstaining. Antibodies included rabbit anti-myelin basic protein (MBP) (Abcam, Cambridge, MA, United States, ab40390, 1:800 dilution), FNB (blue fluorescent Nissl dye, Invitrogen, Carlsbad, CA, United States, N21479, 1:200 dilution), and Alexa Fluor 594 goat antirabbit IgG (Invitrogen, Carlsbad, CA, United States, A11037, 1:1,000 dilution).

We used immunostaining of the immediate early gene *cFos* expression to reflect the neuronal response activity under stress conditions (Chan et al., 1993; Farivar et al., 2004). Whole brain serial slices were first stained with rabbit anti-c-Fos (Synaptic Systems, Goettingen, Germany, #226003, 1:2,000 dilution) and Alexa Fluor 488-conjugated donkey antirabbit IgG (A-21206, Invitrogen, Carlsbad, CA, United States). The slices were then counterstained with the following primary antibodies: mouse anti-PV (Millipore, Billerica, MA, United States, MAB1572, 1:1,000 dilution) and goat anti-ChAT (Millipore, Billerica, MA, United States, AB144P, 1:500 dilution). The following secondary antibodies were used: Alexa Fluor 594-conjugated donkey antigoat IgG and Alexa Fluor 647-conjugated donkey antimouse IgG (A-11058 and A-31571, respectively). All antibodies were used at 1:1,000 dilution and obtained from Invitrogen (Carlsbad, CA, United States).

For the demonstration of batch brain slice registration from the different kinds of samples (Figure 3D), we used one slice from the *in situ* hybridization data from The Allen Brain Atlas^1^.

### Data Registration and Quantitative Analysis

For cell recognition, the images were sequentially filtered with the top-hat operation, binarization, and opening operations. The resolution of the images used in this study was 0.65 μm/pixel; therefore, we chose a disk-shaped structure element with a radius of 9 pixels for the top-hat filter and 5 pixels for the opening filter. For binarization, the threshold depends on the signal-to-background ratio and the absolute intensity of the image, and we selected 30 as the fluorescence intensity threshold.

For slice registration, the atlas was investigated page by page, and the most similar atlas section was selected. The atlas was also rotated to obtain an appropriate degree of slant section if needed. This was achieved by controlling a virtual point in the interface, and the atlas could be rotated along its yaw axis and pitch axis at any angle in real time to obtain a new section in the current atlas pose. Once the atlas section fits the slice sufficiently, the feature points are labeled on the slice image according to the recommended points on the atlas section image. The feature points are usually located at the boundary of obvious brain regions, such as the cortex and fiber tracts. The points depended on the extent of distortion of the brain slice and the accuracy of the study. In this study, 40–60 feature points were labeled per slice, and it took 3–5 min. Once all the point pairs were labeled, the deformation field was calculated based on the thin plate spline (TPS) algorithm (Bookstein, 1989), and the slice image was covered with a deformed atlas for preview; consequently, the operator may verify and modify or save the result.

When all slices were recognized and registered, the number of labeled neurons in each region was counted automatically. All these procedures were based on a graphical operation interface, named Serial Section Registration. It can be found online at http://atlas.brainsmatics.org/a/ssr2021. The imaging processing handbooks may be helpful to follow the above image processing operations, such as Digital Image Processing written by Rafael C. Gonzalez and Richard E. Woods (Gonzalez and Woods, 2008).

All graphs were generated using the Prism v8.0 software (GraphPad, La Jolla, CA, United States), and the data were analyzed with the two-tailed *t*-test using the Prism software. The confidence level (*P*-value) was set to 0.05, and the results were presented as mean ± SEM.

## Results

### An Integrated Pipeline for Processing Batch Tissue Sections

To simplify the processing of complete brain tissue slices, we established a tissue slice batch processing scheme (Figure 1). The program contained continuous tissue section acquisition, batch staining, slice registration, and signal analysis. Using this set of standard procedures, we can quickly achieve efficient processing of batch brain sections.

To obtain serial complete brain slices, we first embedded the mouse brain with agarose and then sectioned it with a vibratome (Figure 1A). Agarose embedding is one of the most utilized methods for obtaining continuous brain slices. Sample embedding has two purposes; one is to give the sample relatively hard support when slicing, and the other is to collect a complete slice in the part of the separated tissue, such as the olfactory bulb and brainstem. We placed collected continuous slices in a staining plate by means of cyclic sequence placement during slices collection, which put each slice in each different hole side by side. This placement method facilitates the rapid extraction of equally spaced brain slices (Figure 1A) and optimizes the use of time and materials for subsequent experiments.

We used the separation of the staining plate and the 24-well plate to replace the cumbersome process of aspiration in each hole (Figure 1B). This reduced a large number of repeated operations in the experiment and resulted in rapid staining. First, using the fitting and separation of the 24-well plate and the staining plate, the rapid processing of batch slices was performed efficiently. This design achieves a single fluid change for all sections and simplifies the steps based on the traditional operation method. Second, the strainer in the staining plate can not only accelerate the penetration speed of the dyeing solution when the two sides of the brain slice contact the solution but also effectively ensure the integrity of the tissue and avoid damage to the brain slice. Finally, to reduce the evaporation of the dyeing solution during the long-term dyeing process, we added a cylinder that fits into the staining plate one by one inside the cover of the entire device to fill the excess space in each hole (Figure 1B). This maintains the humidity of the microenvironment in the entire device, reducing the possibility of drying the brain slices. Moreover, the staining solution stored in the 24-well plate can be repeatedly used for multiple rounds of slice staining, which benefits for increasing the efficiency of using the dye solution. Especially for precious antibodies using, this method maximizes the efficiency of antibodies while ensuring the quality and quantity of staining.

To quantitatively analyze the stained images of batch slices, this study developed two sets of graphical interactive interfaces for cell recognition (Figure 1C left) and registration of brain slice images (Figure 1C middle). During the identification of the cell body or cell nucleus of a neuron, this method first suppresses the background signal of the gray-scale image of the brain slice to make it uniform through the top-hat algorithm. Then, the image is binarized, and the slightly connected cell body or nucleus is segmented through the binary opening operation, or possible nerve fiber signals are eliminated. Finally, we used the region-props function with the MATLAB software (R2021a) to analyze the connected domain of the binary image to obtain the cell body signal.

In summary, we established a set of standard procedures from complete slice acquisition and batch slice rapid staining for data registration to realize the whole brain information acquisition, which simplifies the experimental operation and data analysis.

### Serial Immunochemical Staining of Batch Tissue Sections

**FIGURE 2:**
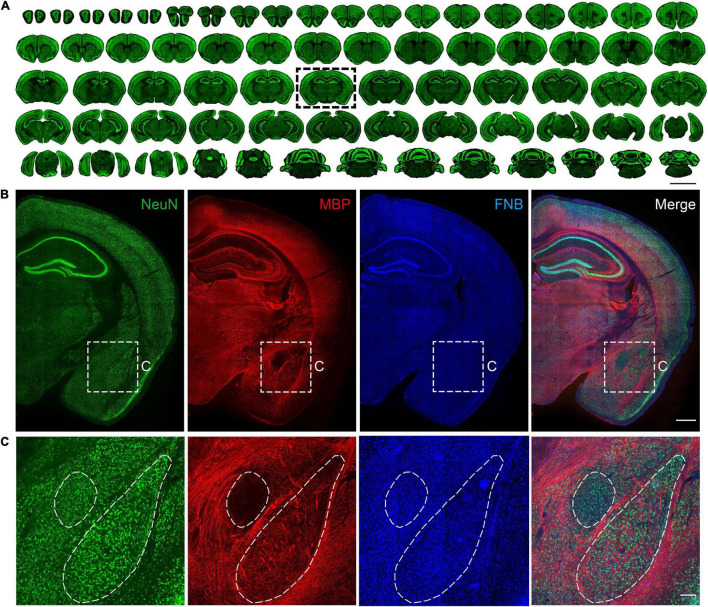
Multiple views of structural information based on immunostaining. **(A)** Maximum intensity projections of the representative coronal planes of immunofluorescence staining of the NeuN in 50 μm thickness in the whole mouse brain. The scale bar is 5 mm. **(B)** Coronal results of the NeuN antibody, MBP antibody, and FNB staining. The scale bar is 1 mm. **(C)** Partially enlarged view of the area is shown in subgraph B. The scale bar is 300 μm.

We implemented batch staining of 50 μm serial slices of the complete brain according to the above scheme. We obtained the staining results of tissue sections of the mouse brain (Figure 2A). As a macromolecule, agarose cannot penetrate the internal structure of the mouse brain, so the slices obtained by this method are almost indistinguishable and are expected to be used for subsequent registration from two-dimensional to three-dimensional structures. An advantage of agarose sample preparation is its compatibility with immunochemical staining. This allows convenient labeling of different cell types or states, enabling cell-type-specific brain connectivity and activity mapping. As a commonly used biomarker for neurons, NeuN can effectively provide information about the spatial distribution of almost all neurons in the brain. From the coronal slices displayed above, we can see that most of the collected slices were morphologically intact, and immunochemical staining of NeuN was uniform (Figure 2A). Overall, 279 intact slices were acquired, and four slices were partially lost. The ratio of intact slices to all slices in this pipeline was 98.5%. For the staining of 279 brain slices in the whole process, antibody incubation requires 27 h, while multiple rinsing of batches of brain slices only requires 1 h, which is equivalent to the immunochemical staining time of a single or a small number of brain slices. This shows that this scheme reduces the time required for multiple repeated liquid exchanges during the batch slicing process and greatly improves the efficiency of the experiment.

Through the abovementioned slice placement sequence, one brain slice in the complete brain series was quickly extracted, and the results of multilabeling of neuronal cells, fibers, and cytoarchitectonic information were obtained (Figure 2B). NeuN provides information regarding the neuron soma. MBP provides information on neuron fibers, and FNB provides information on cytoarchitectonics. In the central amygdala region on the ventral side, neuronal cell bodies were evenly distributed. In this brain area, the nucleus boundary can’t be distinguished by the cell body distribution, while it can be identified by the fiber bundles information (Figure 2C). Although there are few fibers in the basolateral amygdala, the fiber bundles projected from the upper corpus callosum or thalamus are neatly arranged on both sides of the BLA, distinguishing the basolateral amygdala from other areas. This is consistent with the results of several types of mouse brain atlas (George Paxinos, 2001; Dong, 2008). The above results show that this method can counterstain multiple antibodies or dyes while processing slices in batches, which can quickly obtain multiple information of any brain slice.

### The Result of Data Processing for Batch Images

To analyze the image data of stained slices, the biologists need to identify the labeled neuron from the image and register their coordinates to the standard atlas to acquire region-specific information. The traditional method of manually counting or drawing to segment regions is inefficient and unsuitable for batch image data. Some image processing software, such as Photoshop, was also used for slice image deformation, but this software is usually incompatible with customized needs such as cell counting. In addition, cases always occur, such as inconsistent section angles between samples, which results in registration being more difficult. To overcome these difficulties, we developed a data analysis process to recognize fluorescence-labeled neuronal somata and register them to the Allen Mouse Brain Common Coordinate Framework (CCFv3) (Vogt, 2020; Wang et al., 2020).

For the identification of fluorescence-labeled neuronal soma, the background of the slice image was subtracted from the original image to make the image uniform using the top-hat algorithm. Next, the image was binarized with a given threshold to extract the foreground and cell signals. An opening operation was performed to break the slight connection between the adjacent cell body and remove trivial signals. Cell identification was achieved using the connected component analysis (Supplementary Figure 2).

**FIGURE 3:**
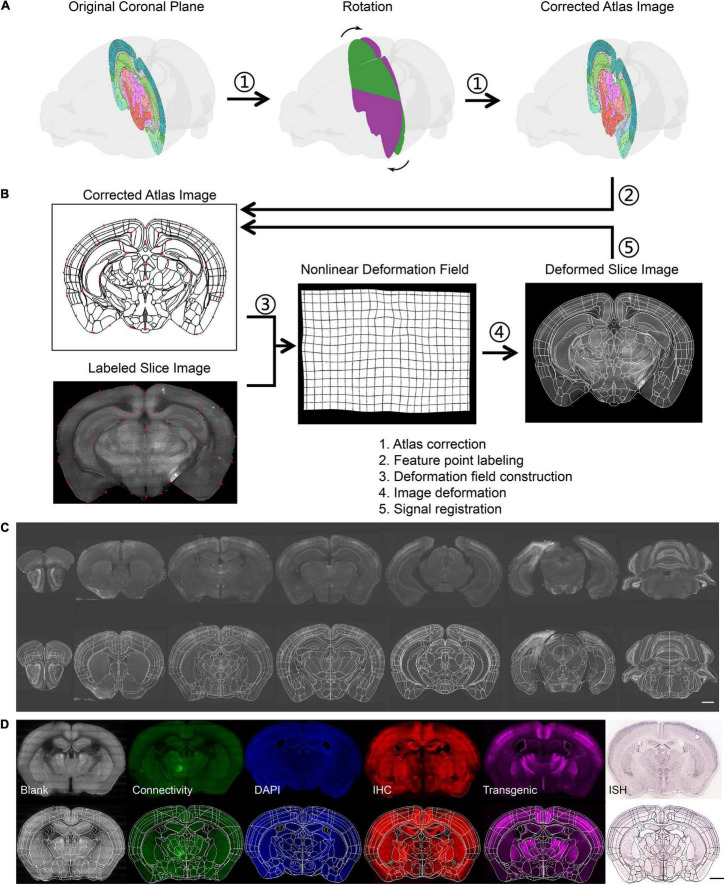
Brain registration procedures. **(A)** The procedure to get the corrected atlas section by rotating the coronal plane to the slant slice plane. **(B)** The registration principle of brain slice and atlas. **(C)** The results of batch brain slice registration from the same sample. **(D)** The results of batch brain slice registration from a different kind of sample. The scale bar is 1 mm.

After segmentation, the identified neurons were assigned to the corresponding brain region by registration between the slice image and atlas section. Because of the inconsistent angle of the brain section in different samples, we rotated the section plane and resliced the 3D atlas to obtain a proper atlas image that was well-matched with the brain slice (Figure 3A and Supplementary Figure 3). Then, the slice image and atlas section were labeled with feature points in a semiautomatic manner (Supplementary Figure 4), that is, the feature points on the atlas would be automatically labeled on the slice image based on affine transformation, so they could be manually adjusted just in adjacent areas. A deformation field was calculated using point pairs based on the TPS algorithm for image registration (Figure 3B). Finally, the locations of identified neurons were mapped into the Allen Mouse Brain CCF to obtain their brain regions; consequently, the regional signal number could be counted. To obtain higher accuracy, some slices with separation or severe deformation were split into multiple parts by manual selection, and each part was registered as a parallel task (Supplementary Figure 5).

Using the method mentioned above, we registered all slice images of one sample to the CCF (Figure 3C). Several slices with similar section planes but distinct staining or labeling methods and from different individuals were also registered (Figure 3D). This indicated that our method was also compatible with the multimodal data.

### Batch Processing of Tissue Sections Revealed the Absence of Learned Despair in Aging Mice

As an example, we investigated the neural circuits involved in stress response under the FST in young and aging mice. In this case, the responding neurons are distributed widely throughout the brain, and the principal area of differences is unknown. An advantage of our strategy is its high-throughput profiling of brain-wide cellular phenotypes. This allows for the convenient mapping of cell-type-specific neurons in the entire brain. Therefore, we used a strategy to detect the different neuronal response activity under stress conditions by immunostaining for the immediate early gene *cFos* expression.

**FIGURE 4:**
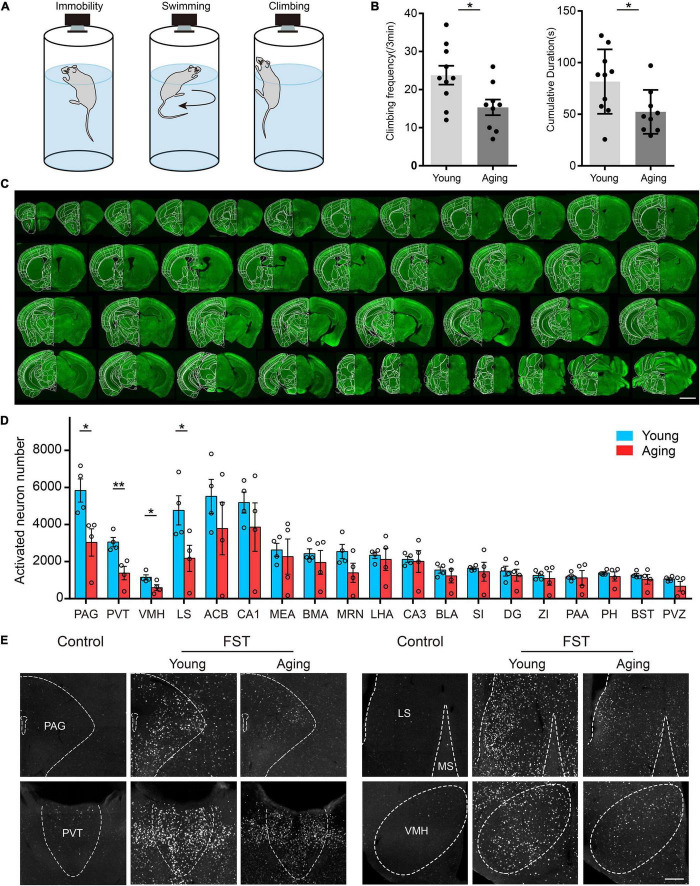
c-Fos activation in young and aging mouse brains in response to forced swimming stress. **(A)** Three behavior postures of the mouse in FST, namely, immobility, swimming, and climbing. **(B)** The aging mice showed a significant reduction in the number and time of climbing in the FST. For all panels, *n* = 9 per group, * indicates false discovery group (FDR) *q*-value < 0.05, based on *P*-values from unpaired two-sample *t*-test comparing the young group to the aging group. **(C)** A large number of neuron signals appeared in many cortical and subcortical areas. **(D)** The analysis of c-Fos activation in multiple subcortical brain areas. For all panels, *n* = 4 per group, * indicates false discovery group (FDR) *q*-value < 0.05, ** indicates FDR *q*-value < 0.01, based on *P*-values from unpaired two-sample *t*-test comparing the young group to the aging group. Scatter points represent the c-Fos activation of each animal. Error bars, mean ± SEM. **(E)** c-Fos activation was significantly reduced in the PAG, PVT, VMH, and LS in aging mice. The scale bars are for **(C)** 2 mm and for **(E)** 200 μm.

Eighteen C57 mice were tested in the FST experiment, nine of which at 2–3 months were a normal adult group, and nine mice at 20 months belonged to the aging group. In addition, another two adult mice were selected as the control group without FST. In the FST, we divided the activity state of the mice into three categories, namely, immobility, swimming, and climbing (Figure 4A). While the mice were forced to swim, the behavioral software on the computer recorded and analyzed the activities of the mice in real time. The results showed that the number and time of climbing exercises in the aging group were significantly reduced compared with those in the normal adult group (Figure 4B). The mean sensitivity of the aging group under stress was reduced. To detect the activity and response of neurons, animals were sacrificed at 1.5 h after FST (i.e., the control group was in the corresponding resting state) for c-Fos staining of batch sections. We observed that many neuron signals appeared in multiple cortical and subcortical areas of all tested mice, which indicated that the c-Fos protein was expressed in these brain areas (Figure 4C). Consistent with previous studies (Cryan et al., 2005; Choi et al., 2013; Wang et al., 2019), various regions containing abundant c-Fos-positive neurons make a great contribution to the FST, such as the medial prefrontal cortex (mPFC), lateral septal nucleus (LS), paraventricular nucleus of the thalamus (PVT), and paraventricular nucleus of the hypothalamus. Then, we counted all c-Fos-positive neurons using our registration and automatic counting methods. We found that several areas of the aging group presented a weak activity in the FST compared with the normal adult group, including the periaqueductal grey (PAG), PVT, ventromedial hypothalamic nucleus (VMH), and LS (Figures 4D,E). This suggests that these brain areas in aging mice are more related to their manifestations of depression under stress conditions. Furthermore, with our scheme, we achieved multicolor counterstaining of parvalbumin (PV) and cholinergic (ChAT) neurons to detect the cell types in some brain areas in FST mice.

**FIGURE 5:**
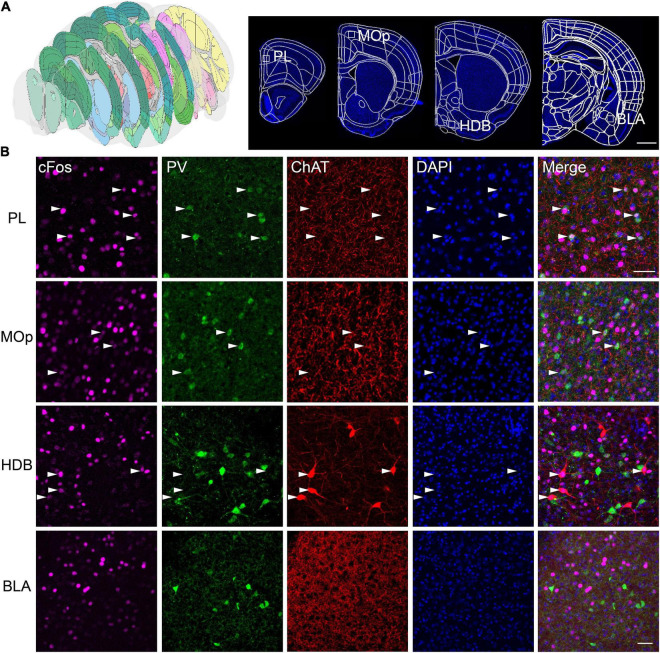
Multicolor counterstaining indicates that PV and ChAT neurons in some brain areas participate in the neural circuit response in FST. **(A)** The location of the selected slices. **(B)** PV+ neurons in the PL, the primary motor cortex (MOp), and ChAT+ neurons in the horizontal limb of the diagonal band (HDB) are merged with c-Fos in FST. White arrowheads indicate c-Fos+/PV+, and c-Fos+/ChAT+ neurons. Scale bars: **(A)** 1 mm and **(B)** 100 μm.

We selected typical coronal planes in the complete brain (Figure 5A). The results show that almost all the PV+ neurons in the prelimbic (PL) were found to be activated in the FST, but, primarily, non-PV neurons in the nucleus of the horizontal limb of the diagonal band (HDB), which contains many PV neurons (Figure 5B). Thus, the forced swimming stress primarily activated PV neurons in the PL and ChAT neurons in the HDB, suggesting that the PV neurons in the HDB may have different functions from those in the PL. These results also demonstrate the effectiveness of our scheme in combining fast fluorescence imaging with antibody staining to investigate the active roles of specific cell types in brain function.

## Discussion

Mapping the neuronal location and activity throughout the different regions of the brain, even for a complete brain, can be valuable for the discovery of neural circuit organization, but labor and low throughput have been significant obstacles to performing unbiased screening. This limits the serial experiments in multiple samples. In this study, we developed a simple solution for batch processing of brain slices. Combined with the actual case of immunochemical staining, we demonstrated the detailed process of using this scheme to achieve large-scale staining, registration, and signal quantitative calculation of batch slices. This method is suitable for tissue slices obtained by vibrating sections and can be used for repeated staining of different cellular phenotypes, which are relatively easy to operate and may provide a great convenience for scientific researchers.

Compared with traditional methods (Mathieu et al., 2014; Chang and Kawai, 2018), the present processing scheme uses the device of the utility model to simplify the manual and repeated operations in the batch processing of slices. Overall, 283 intact slices were acquired, and four slices were partially lost (Figure 2A). The ratio of intact slices to all slices in this pipeline was 98.5%. It reduces the damage rate of brain slices to <5% and improves the efficiency of the antibody. This simple method is feasible for processing animal tissue samples, such as vibrating sections, frozen sections, and paraffin sections. In addition to immunochemical staining, it can also be applied to pathological staining and *in situ* hybridization experiments (Figure 3D). The homogeneity and stability of this method for different sections and staining experiments were tested. Because the deformation of brain slices is small during the entire operation, the data obtained from the batch slice processing are expected to be used for three-dimensional reconstruction and registration (Gleave et al., 2013; Yan et al., 2018). Meanwhile, the transition from mice to non-human primates faces the challenge of multiplying the area of tissue slices due to the continuous upgrading of model animals (Boldog et al., 2018; Zhao et al., 2020). For the staining of large-volume sample tissue sections, complications such as significant time consumption and difficulty in realizing uniform staining are routinely introduced (Kohl et al., 2014; Na et al., 2020). Therefore, improving the device of the utility model to fit large sample tissue sections is expected to speed up the staining efficiency, which can promote the structural and functional analysis of large samples.

In addition, we provided a simple but acceptable approach to identify the fluorescence-labeled neuronal soma or nucleus. It was achieved only with the morphological operation and binarization, which appears similar to the ImageJ software. However, it can be combined with registration. Compared with some complex algorithms such as the deep-learning-based method (Falk et al., 2019; Stringer et al., 2021), our segmentation method treats the signals as spots and ignores the fine structure of neuron-like dendrites. It would also be difficult to segment adjacent cells with a low threshold, leading to the dilemma that the cells with lower activation were excluded, which could have provided valuable biological information in this experiment. Therefore, the image enhancement algorithm can be used to improve the signal-to-background ratio. Nevertheless, our method provided a uniform standard to avoid personal bias in cell identification. Furthermore, it helps to satisfy a basic but general need to search for areas with significant differences in cell numbers.

In recent years, typical 3D imaging and registration methods have been developed, such as micro-optical sectioning tomography (Gong et al., 2016; Ni et al., 2021) and series two-photon tomography (Amato et al., 2016; Wang et al., 2020) and whole-brain clearing techniques combined with light-sheet microscopy (Renier et al., 2016; Fürth et al., 2018), to collect three-dimensional continuous brain imaging data, as well as the relevant 3D registration method. These methods have incomparable advantages in reconstructing long-range projections and studying the diversity of neuron morphology. However, limitations such as complicated specimen preparation and expensive imaging equipment have prevented these methods from spreading. 2D brain slices acquired with a vibratome or freezing microtome are still widely applied. In addition, for studies on cell number counting or gene expression analysis, the 2D image could also provide the information. The problems did exist that the different deformations between consecutive slices made it difficult to reconstruct the 2D slice into the 3D brain and register with 3D registration methods. An approach based on manually labeling feature points on 2D slices has been reported (Zhang et al., 2016), which was similar to our study. But we adopted a non-linear deformation model rather than merely affine deformation to revise the local distortion. There was also a strategy that utilized the automatically extracted contour feature points for non-linear TPS deformation (Fürth et al., 2018). However, the information provided by the slice contour is limited and easily impacted by the brain slice tear or breakage. Instead, manually labeled feature points or extracted points using the SURF algorithm with the internal structural features of brain slices could highlight the inner anatomical feature, which has a higher tolerance and compatibility toward the slice with different deformations and modalities (Song et al., 2020). The deep-learning network might be a feasible approach to extract the feature points on slice images, and more research is merited.

In conclusion, this study established a simple method for batch processing of tissue slices, which is convenient for researchers in the field of life science. The staining of tissue sections and the acquisition of whole brain information are helpful for systematically studying the structure and function of brain neural networks and promoting the development of related research such as brain diseases.

## Data Availability Statement

The original contributions presented in the study are included in the article/Supplementary Material, further inquiries can be directed to the corresponding authors.

## Ethics Statement

All animal experiments were approved by the Animal Ethics Committee of the Huazhong University of Science and Technology.

## Author Contributions

HG and XL conceived and designed the study. SC and BL performed the experiments. SC, AL, and ZL performed the data analysis. XL, BL, SC, and ZL prepared the figures and wrote the manuscript. All authors contributed to the article and approved the submitted version.

## Conflict of Interest

The authors declare that the research was conducted in the absence of any commercial or financial relationships that could be construed as a potential conflict of interest.

## Publisher’s Note

All claims expressed in this article are solely those of the authors and do not necessarily represent those of their affiliated organizations, or those of the publisher, the editors and the reviewers. Any product that may be evaluated in this article, or claim that may be made by its manufacturer, is not guaranteed or endorsed by the publisher.

## Footnotes

1
http://www.brain-map.org

## References

[B1] Amato S. P. Pan F. Schwartz J. Ragan T. M. (2016). Whole brain imaging with serial two-photon tomography. *Front. Neuroanat.* 10:31. 10.3389/fnana.2016.00031 27047350 PMC4802409

[B2] Boldog E. Bakken T. E. Hodge R. D. Novotny M. Aevermann B. D. Baka J. (2018). Transcriptomic and morphophysiological evidence for a specialized human cortical GABAergic cell type. *Nat. Neurosci.* 21 1185–1195. 10.1038/s41593-018-0205-202 30150662 PMC6130849

[B3] Bookstein F. L. (1989). Principal warps: thin-plate splines and the decomposition of deformations. *IEEE Trans. Pattern Anal. Mach. Intell.* 11 567–585. 10.1109/34.24792

[B4] Cai R. Pan C. Ghasemigharagoz A. Todorov M. I. Forstera B. Zhao S. (2019). Panoptic imaging of transparent mice reveals whole-body neuronal projections and skull-meninges connections. *Nat. Neurosci.* 22 317–327. 10.1038/s41593-018-0301-303 30598527 PMC6494982

[B5] Castagné V. Moser P. Roux S. Porsolt R. D. (2011). Rodent models of depression: forced swim and tail suspension behavioral despair tests in rats and mice. *Curr. Protoc. Neurosci.* Chapter 8:Unit 8.10A. 10.1002/0471142301.ns0810as55. 21462162

[B6] Chan R. K. Brown E. R. Ericsson A. Kovács K. J. Sawchenko P. E. (1993). A comparison of two immediate-early genes, c-fos and NGFI-B, as markers for functional activation in stress-related neuroendocrine circuitry. *J. Neurosci.* 13 5126–5138. 10.1523/JNEUROSCI.13-12-05126.1993 8254363 PMC6576398

[B7] Chang M. Kawai H. D. (2018). A characterization of laminar architecture in mouse primary auditory cortex. *Brain Struct. Funct.* 223 4187–4209. 10.1007/s00429-018-1744-1748 30187193

[B8] Choi S. H. Chung S. Cho J. H. Cho Y. H. Kim J. W. Kim J. M. (2013). Changes in c-Fos expression in the forced swimming test: common and distinct modulation in rat brain by desipramine and citalopram. *Korean J. Physiol. Pharmacol.* 17 321–329. 10.4196/kjpp.2013.17.4.321 23946692 PMC3741489

[B9] Craddock R. C. Jbabdi S. Yan C.-G. Vogelstein J. T. Castellanos F. X. Di Martino A. (2013). Imaging human connectomes at the macroscale. *Nat. Methods* 10 524–539. 10.1038/nmeth.2482 23722212 PMC4096321

[B10] Cryan J. F. Valentino R. J. Lucki I. (2005). Assessing substrates underlying the behavioral effects of antidepressants using the modified rat forced swimming test. *Neurosci. Biobehav. Rev.* 29 547–569. 10.1016/j.neubiorev.2005.03.008 15893822

[B11] Dong H. (2008). *The Allen Reference Atlas: A Digital Color Brain Atlas of the C57Bl/6J Male Mouse.* New Jersey, NJ: John Wiley & Sons.

[B12] Economo M. N. Clack N. G. Lavis L. D. Gerfen C. R. Svoboda K. Myers E. W. (2016). A platform for brain-wide imaging and reconstruction of individual neurons. *eLife* 5:e10566. 10.7554/eLife.10566 26796534 PMC4739768

[B13] Falk T. Mai D. Bensch R. Çiçek Ö Abdulkadir A. Marrakchi Y. (2019). U-Net: deep learning for cell counting, detection, and morphometry. *Nat. Methods* 16 67–70. 10.1038/s41592-018-0261-262 30559429

[B14] Farivar R. Zangenehpour S. Chaudhuri A. (2004). Cellular-resolution activity mapping of the brain using immediate-early gene expression. *Front. Biosci.* 9 104–109. 10.2741/1198 14766350

[B15] Fitzgerald P. J. Hale P. J. Ghimire A. Watson B. O. (2020). The cholinesterase inhibitor donepezil has antidepressant-like properties in the mouse forced swim test. *Transl. Psychiatry* 10:255. 10.1038/s41398-020-00928-w 32712627 PMC7382650

[B16] Fürth D. Vaissière T. Tzortzi O. Xuan Y. Märtin A. Lazaridis I. (2018). An interactive framework for whole-brain maps at cellular resolution. *Nat. Neurosci.* 21 139–149. 10.1038/s41593-017-0027-27 29203898 PMC5994773

[B17] George Paxinos K. F. (2001). *The Mouse Brain in Stereotaxic Coordinates.* London: Academic Press.

[B18] Gleave J. A. Lerch J. P. Henkelman R. M. Nieman B. J. (2013). A method for 3D immunostaining and optical imaging of the mouse brain demonstrated in neural progenitor cells. *PLoS One* 8:e72039. 10.1371/journal.pone.0072039 23936537 PMC3735582

[B19] Gong H. Xu D. Yuan J. Li X. Guo C. Peng J. (2016). High-throughput dual-colour precision imaging for brain-wide connectome with cytoarchitectonic landmarks at the cellular level. *Nat. Commun.* 7:12142. 10.1038/ncomms12142 27374071 PMC4932192

[B20] Gonzalez R. C. Woods R. E. (2008). *Digital Image Processing* 3rd Edn. New Jersey, NJ: Pearson Education.

[B21] Helmstaedter M. (2013). Cellular-resolution connectomics: challenges of dense neural circuit reconstruction. *Nat. Methods* 10 501–507. 10.1038/nmeth.2476 23722209

[B22] Hu H. Cui Y. Yang Y. (2020). Circuits and functions of the lateral habenula in health and in disease. *Nat. Rev. Neurosci.* 21 277–295. 10.1038/s41583-020-0292-294 32269316

[B23] Jiang T. Long B. Gong H. Xu T. Li X. Duan Z. (2017). A platform for efficient identification of molecular phenotypes of brain-wide neural circuits. *Sci. Rep.* 7:13891. 10.1038/s41598-017-14360-14366 PMC5654830 29066836

[B24] Kohl J. Ng J. Cachero S. Ciabatti E. Dolan M.-J. Sutcliffe B. (2014). Ultrafast tissue staining with chemical tags. *Proc. Natl. Acad. Sci. U S A.* 111 E3805–E3814. 10.1073/pnas.1411087111 25157152 PMC4246963

[B25] Mathieu E. Benmlih K. Fabre R. Hemmerle J. (2014). An optimized device for staining electron microscopy grids. *Biotech. Histochem.* 89 66–74. 10.3109/10520295.2013.820841 23962218

[B26] Na M. Kim K. Lim H. R. Ha C. M. Chang S. (2020). Rapid immunostaining method for three-dimensional volume imaging of biological tissues by magnetic force-induced focusing of the electric field. *Brain Struct. Funct.* 226 1–13. 10.1007/s00429-020-02160-2160 33175320

[B27] Ni H. Feng Z. Guan Y. Jia X. Chen W. Jiang T. (2021). DeepMapi: a fully automatic registration method for mesoscopic optical brain images using convolutional neural networks. *Neuroinformatics* 19 267–284. 10.1007/s12021-020-09483-9487 32754778 PMC8004526

[B28] Oh S. W. Harris J. A. Ng L. Winslow B. Cain N. Mihalas S. (2014). A mesoscale connectome of the mouse brain. *Nature* 508 207–214. 10.1038/nature13186 24695228 PMC5102064

[B29] Pan C. Cai R. Quacquarelli F. P. Ghasemigharagoz A. Lourbopoulos A. Matryba P. (2016). Shrinkage-mediated imaging of entire organs and organisms using uDISCO. *Nat. Methods* 13 859–867. 10.1038/nmeth.3964 27548807

[B30] Park Y. G. Sohn C. H. Chen R. McCue M. Yun D. H. Drummond G. T. (2018). Protection of tissue physicochemical properties using polyfunctional crosslinkers. *Nat. Biotechnol.* 37 73–83. 10.1038/nbt.4281 30556815 PMC6579717

[B31] Prevedel R. Yoon Y.-G. Hoffmann M. Pak N. Wetzstein G. Kato S. (2014). Simultaneous whole-animal 3D imaging of neuronal activity using light-field microscopy. *Nat. Methods* 11 727–730. 10.1038/nmeth.2964 24836920 PMC4100252

[B32] Qi Y. Yu T. Xu J. Wan P. Ma Y. Zhu J. (2019). FDISCO: advanced solvent-based clearing method for imaging whole organs. *Sci. Adv.* 5:eaau8355. 10.1126/sciadv.aau8355 30746463 PMC6357753

[B33] Ragan T. Kadiri L. R. Venkataraju K. U. Bahlmann K. Sutin J. Taranda J. (2012). Serial two-photon tomography for automated ex vivo mouse brain imaging. *Nat. Methods* 9 255–258. 10.1038/nmeth.1854 22245809 PMC3297424

[B34] Renier N. Adams E. L. Kirst C. Wu Z. Azevedo R. Kohl J. (2016). Mapping of brain activity by automated volume analysis of immediate early genes. *Cell* 165 1789–1802. 10.1016/j.cell.2016.05.007 27238021 PMC4912438

[B35] Song J. H. Choi W. Song Y. H. Kim J. H. Jeong D. Lee S. H. (2020). Precise mapping of single neurons by calibrated 3D reconstruction of brain slices reveals topographic projection in mouse visual cortex. *Cell Rep.* 31:107682. 10.1016/j.celrep.2020.107682 32460016

[B36] Stringer C. Wang T. Michaelos M. Pachitariu M. (2021). Cellpose: a generalist algorithm for cellular segmentation. *Nat. Methods* 18 100–106. 10.1038/s41592-020-01018-x 33318659

[B37] van der Loos C. M. (2007). Multiple immunoenzyme staining: methods and visualizations for the observation with spectral imaging. *J. Histochem. Cytochem.* 56 313–328. 10.1369/jhc.2007.950170 18158282 PMC2326109

[B38] Vogt N. (2020). The mouse reference brain in 3D. *Nat. Methods* 17 655–655. 10.1038/s41592-020-0899-894 32616936

[B39] Wang H. Zhu Q. Ding L. Shen Y. Yang C.-Y. Xu F. (2019). Scalable volumetric imaging for ultrahigh-speed brain mapping at synaptic resolution. *Natl. Sci. Rev.* 6 982–992. 10.1093/nsr/nwz053 34691959 PMC8291554

[B40] Wang Q. Ding S.-L. Li Y. Royall J. Feng D. Lesnar P. (2020). The allen mouse brain common coordinate framework: a 3D reference atlas. *Cell* 181 936–953.e20. 10.1016/j.cell.2020.04.007. 32386544 PMC8152789

[B41] Weyer A. Schilling K. (2003). Developmental and cell type-specific expression of the neuronal marker NeuN in the murine cerebellum. *J. Neurosci. Res.* 73 400–409. 10.1002/jnr.10655 12868073

[B42] Yan H. Pang P. Chen W. Zhu H. Henok K. A. Li H. (2018). The lesion analysis of cholinergic neurons in 5XFAD mouse model in the three-dimensional level of whole brain. *Mol. Neurobiol.* 55 4115–4125. 10.1007/s12035-017-0621-624 28597200

[B43] Zhang S. Xu M. Chang W.-C. Ma C. Hoang, Do J. P. (2016). Organization of long-range inputs and outputs of frontal cortex for top-down control. *Nat. Neurosci.* 19 1733–1742. 10.1038/nn.4417 27749828 PMC5127741

[B44] Zhanmu O. Zhao P. Yang Y. Yang X. Gong H. Li X. (2019). Maintenance of fluorescence during paraffin embedding of fluorescent protein-labeled specimens. *Front. Neurosci.* 13:752. 10.3389/fnins.2019.00752 31396038 PMC6664058

[B45] Zhao S. Todorov M. I. Cai R. Maskari R. A. Steinke H. Kemter E. (2020). Cellular and molecular probing of intact human organs. *Cell* 180 796–812.e19. 10.1016/j.cell.2020.01.030. 32059778 PMC7557154

[B46] Zhong Q. Li A. Jin R. Zhang D. Li X. Jia X. (2021). High-definition imaging using line-illumination modulation microscopy. *Nat Methods* 18 309–315. 10.1038/s41592-021-01074-x 33649587

