# Supplementary material for: High-Throughput Strategy for Profiling Sequential Section With Multiplex Staining of Mouse Brain
Source: Front Neuroanat. 2021 Dec 23;15:771229. doi: 10.3389/fnana.2021.771229 (PMC8732995; doi:10.3389/fnana.2021.771229)
Supplement: Supplementary file 1 [file Data_Sheet_1.docx]

# Supplementary figures


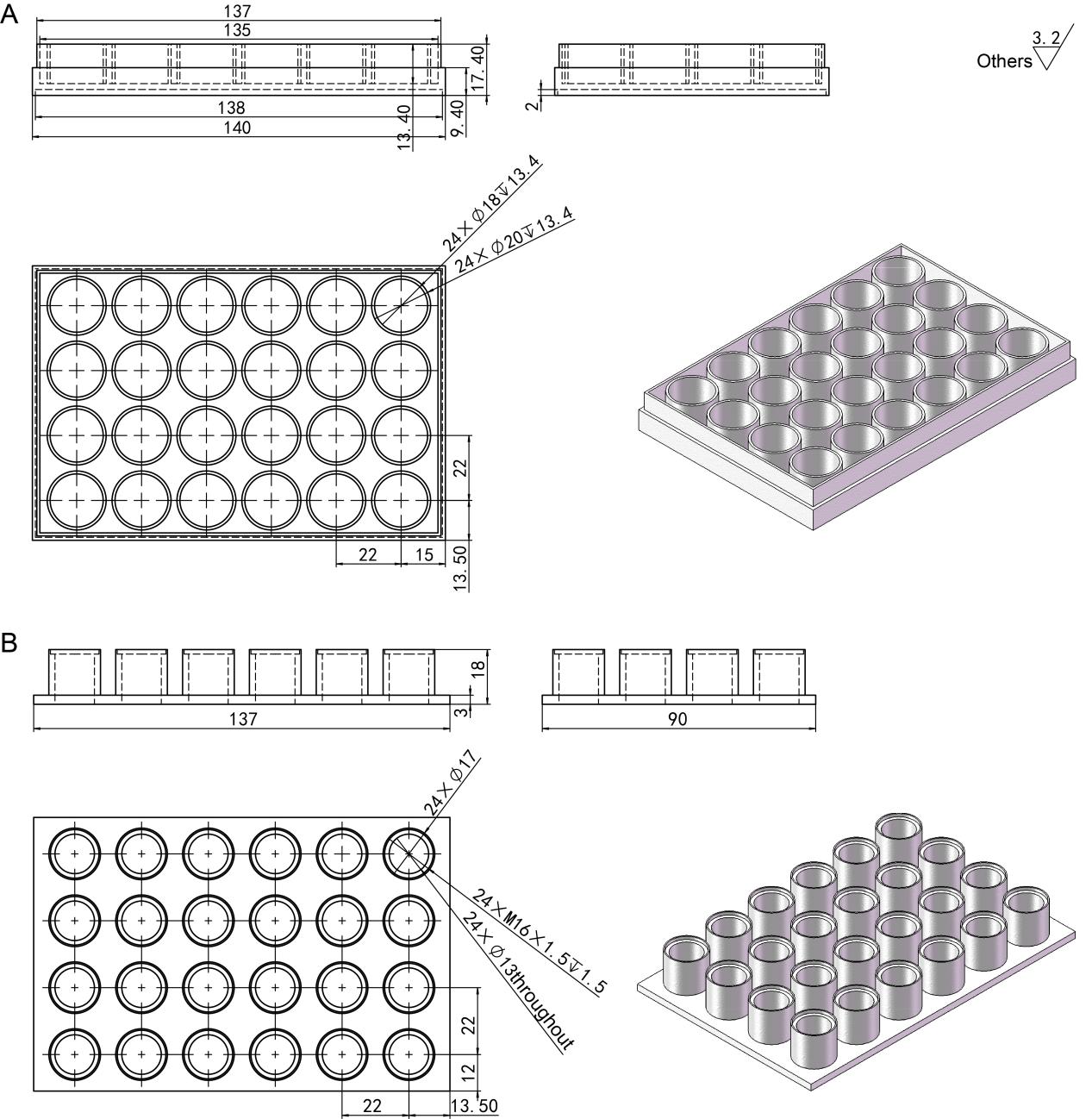


**Figure S1**. Engineering drawing of the 24-well plate system. **(A)** The detailed information of a 24-well plate box for storing dye solution (Unit: mm). **(B)** The detailed information of a 24-well staining plate for placing brain slices (Unit: mm).


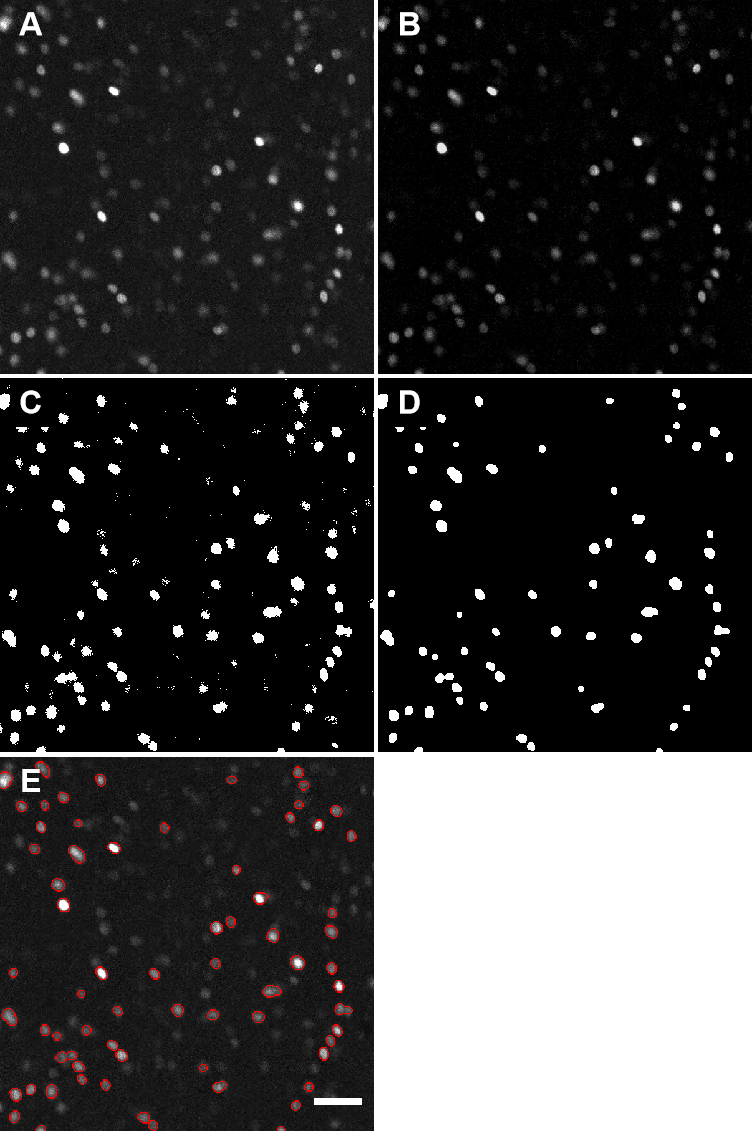


**Figure S2**. The image processing progress for cell recognition. **(A)** The original slice image. The spots with high intensity are the c-Fos expressing neurons. **(B)** The result was obtained by subtracting the background from image in A, using Top-Hat algorithm. The structure element used here is a disk-shape element with a radius of 9 pixels. **(C)** The binarised image in B, with a threshold value as 30. **(D)** The de-noised image in C obtained by holes filling and open operation. The structure element used here is a disk-shape element with a radius of 5 pixels. **(E)** After the connected component analysis of image in D, the identified c-Fos expressing cells were labelled with red circle. Scale bars: 50 μm.


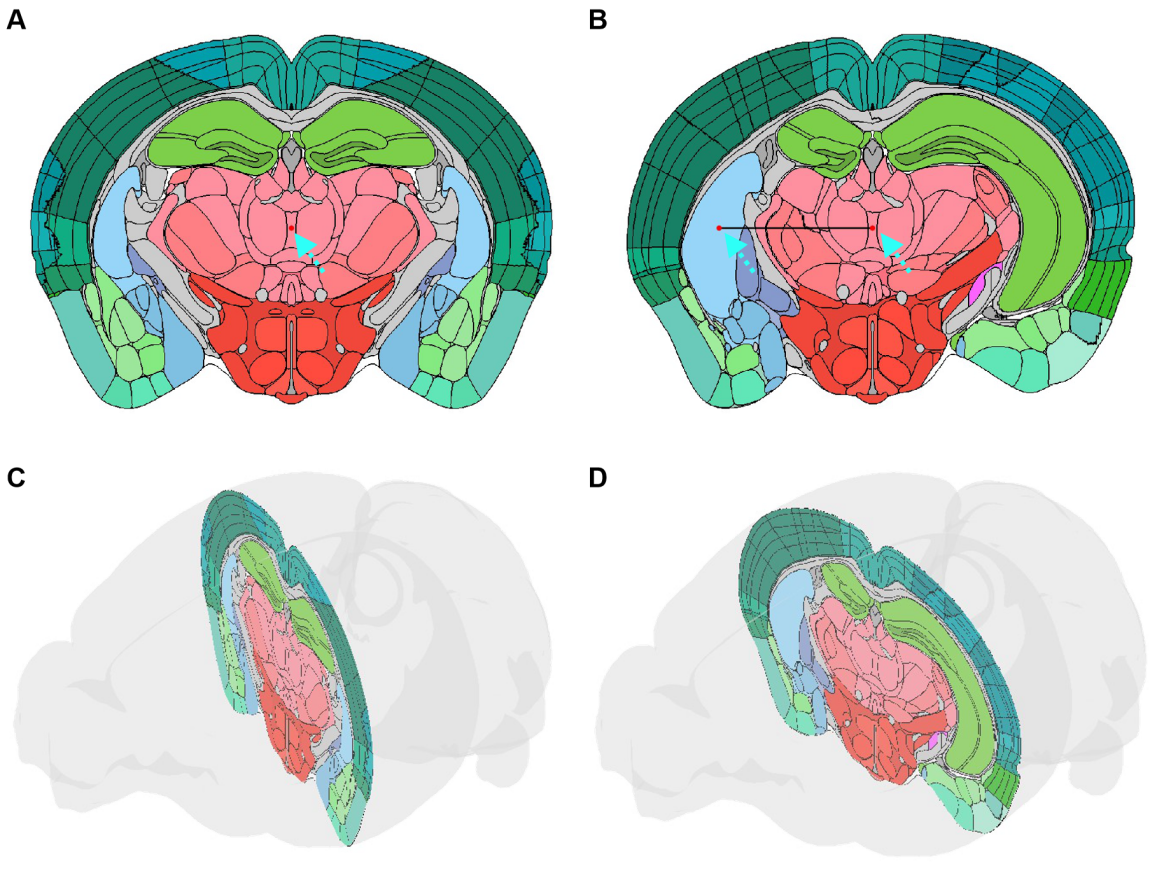


**Figure S3**. The atlas section with any slant angle. The initial atlas section was the standard coronal plane section. The 2D image was shown in A and the current section pose in 3D mouse brain model was shown in C. The red point at the middle of A and indicated with the cyan arrows is the virtual control point. By dragging this point to the destination, the user could obtain a new 2D image of slant atlas section in interface to fit with the real brain slice. The pose of slant section was shown in D.


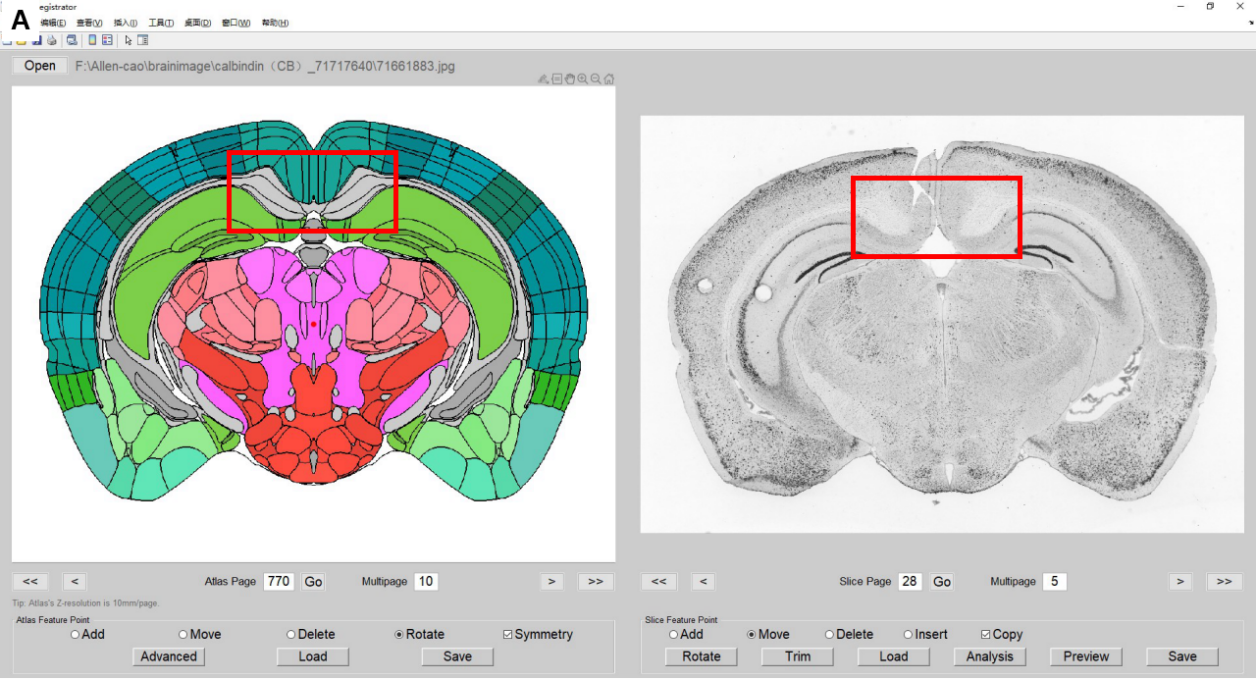


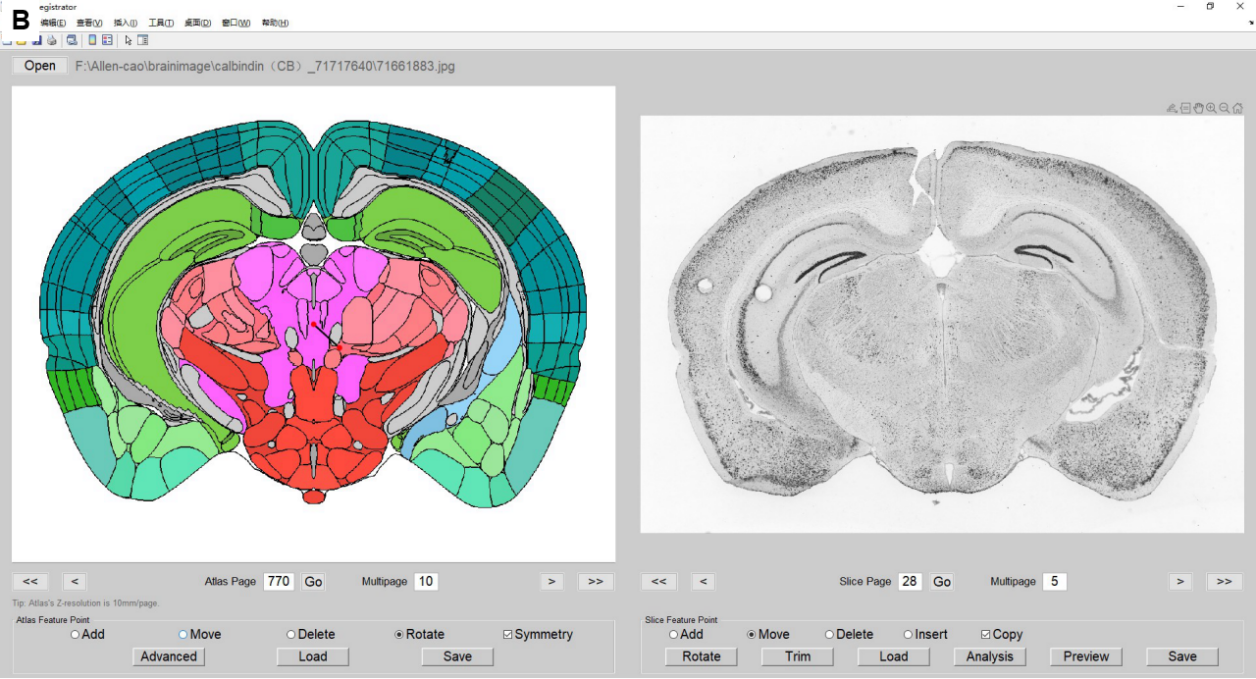


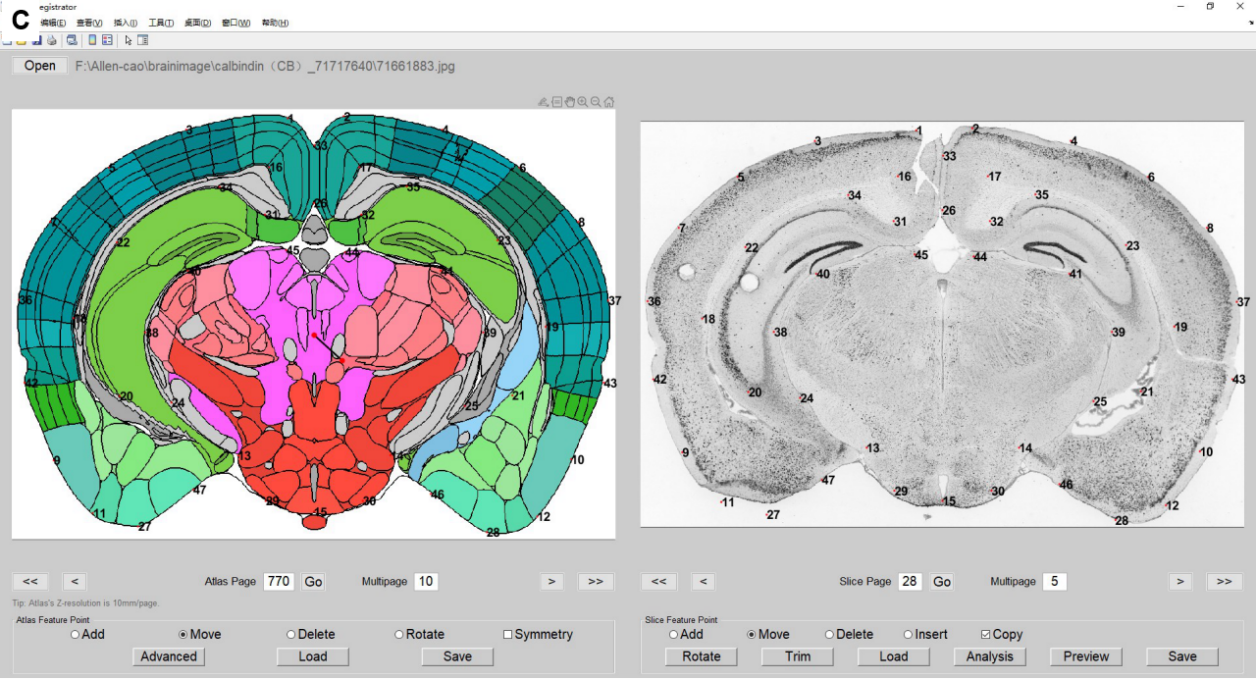


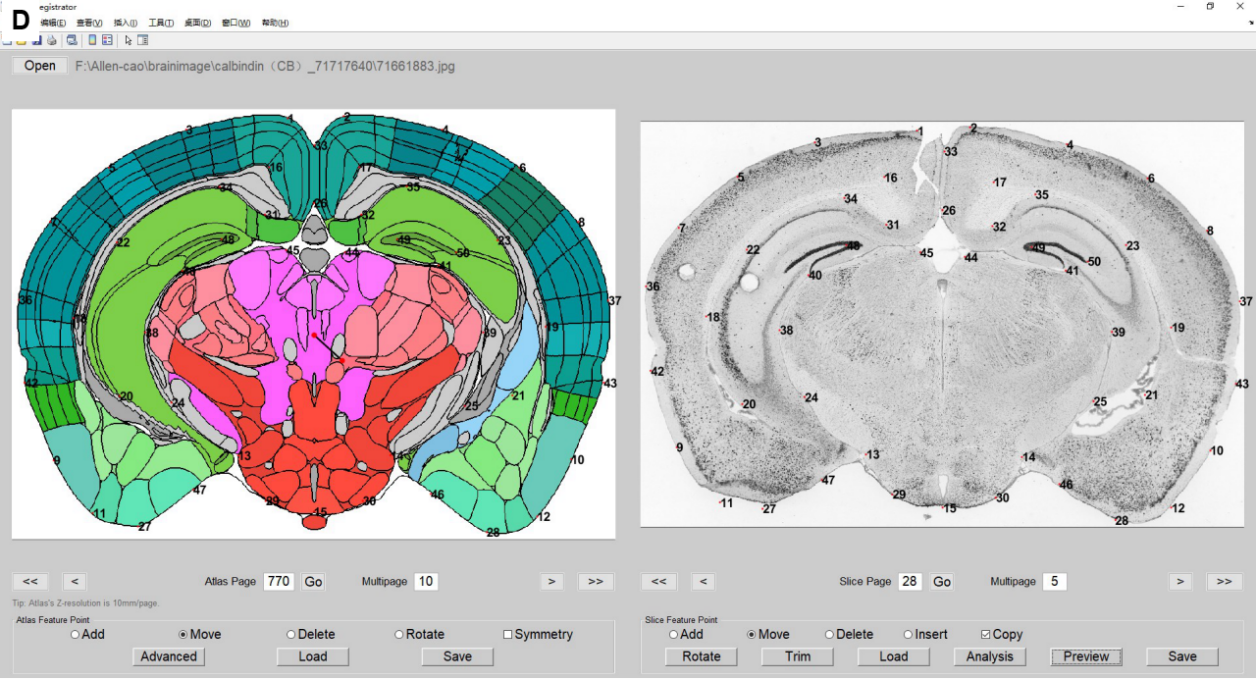


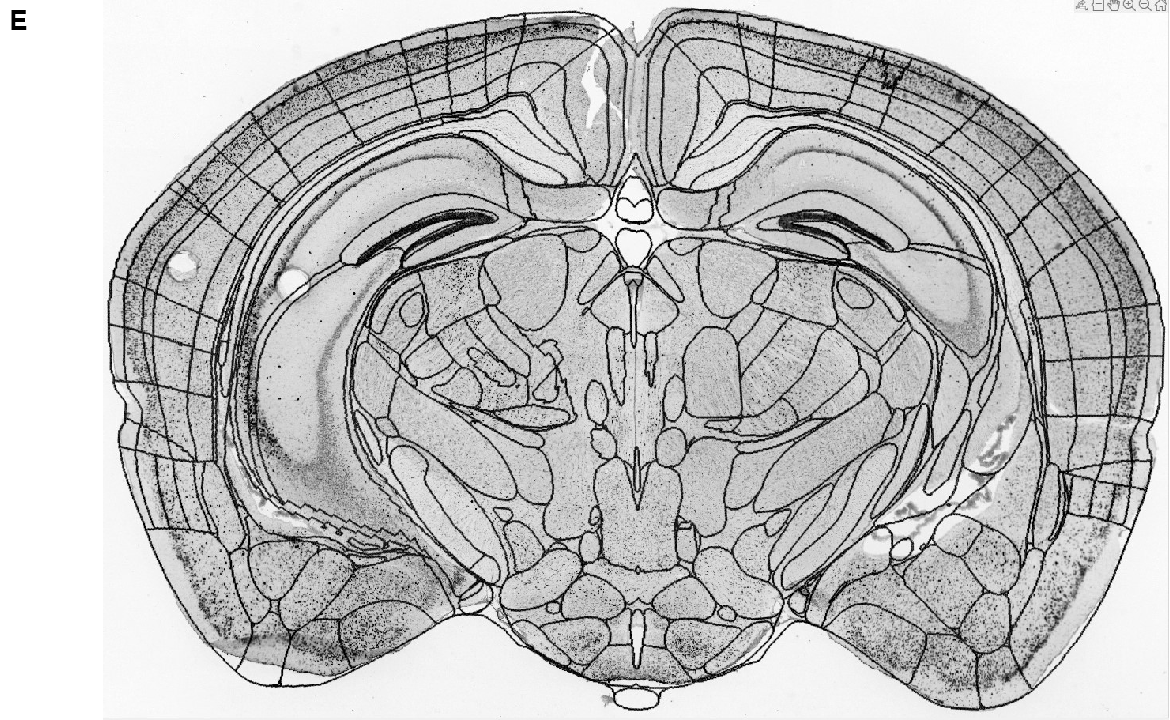


**Figure S4**. The progress of semi-automatic registration. **(A)** The slice image downloaded from the Allen ISH Database as a demo was loaded in the interface. And the most similar atlas coronal plane section was chosen basing on observation of the brain slice image. Because of the slant section, the slice was not horizontally symmetrical, obviously reflected by the hippocampus. In addition, the callosum of two hemispheres were not connected (indicated with the red box) that was different from the standard coronal plane, which indicated the current atlas section was not suitable. **(B)** The atlas section was adjusted by a dragging operation. **(C)** The slice image was rotated and trimmed, and the pre-stored atlas feature points were copied and pasted to the slice image at a ratio. These steps served as affine transformation (rotation, translation, and scaling). **(D)** The feature points of slice in C were finely moved to fit the image feature, with priority on defining image boundaries (e.g. the point 20 for slice was obviously moved to fit the outline of hippocampus), and some more point pairs were added after deformation preview and was followed by reviewing for modification (e.g. the point pairs 48-50 located at the granule cell layer of dentate gyrus). **(E)** The registration result was shown as the original slice image covered with deformed atlas profile.


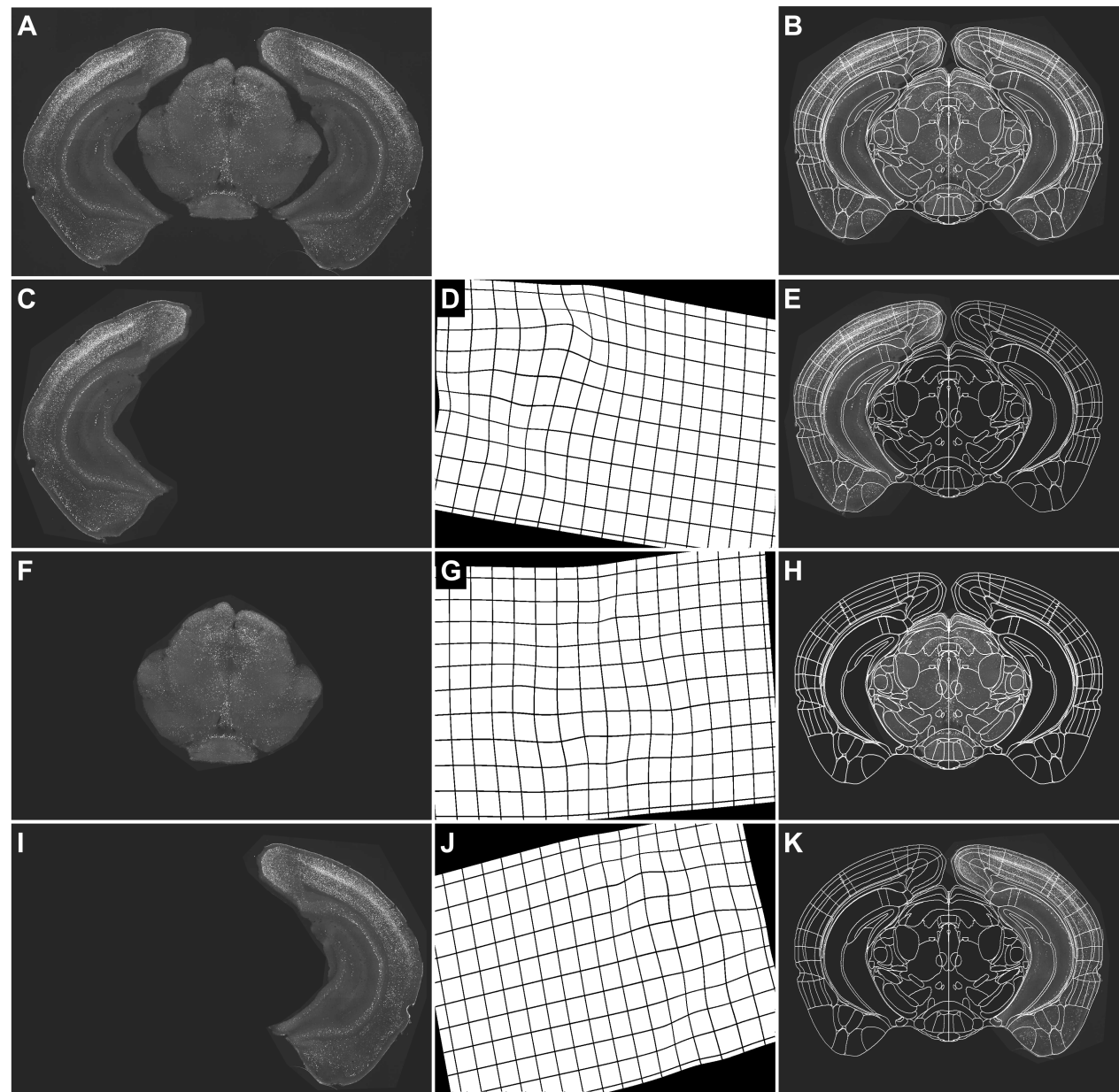


**Figure S5**. The registration of a split-brain slice. **(A)** A typical split slice was shown, whose hippocampus and posterolateral cortex were dramatically isolated from the midbrain, probably caused by the manual operation. **(B)** The final registration effect as the combination of E, H, K. **(C-K)** The slice image in panel A was segmented by manual ROI selection to get three parts. These three images were registered parallelly, whose deformation fields were presented in D, G, J and the registration result were shown in E, H, K.
